# A Case of Calculus, Attended with a Fungus of the Bladder

**Published:** 1782

**Authors:** 


II. *A Case of Calculus, attended with a fungus of the bladder. Communicated by* F. Swediar, M.D. *Physician in London. Read* April 8, 1782.

A Gentleman who had enjoyed a pretty good ſtate of health till about his fortieth year, upon jumping one day out of a phaeton, was ſeized with a moſt acute pain in his back and about the region of the bladder. A few minutes after this, feeling an inclination to make water, he voided with great difficulty and pain a few drops of bloody urine, and the pain increaſing, his urine became totally ſuppreſſed. Repeated venæſections, clyſters, gentle evacuants, the warm bath and other remedies removed theſe alarming ſymptoms in a ſhort time and the natural flow of urine was reſtored. From that time, however, his urine conſtantly afforded more or leſs of a mucous ſediment, and any little irregularity in diet, or violent exerciſe, brought on pain and difficulty in making water, and ſometimes a total ſuppreſſion of urine which more than once brought his life into danger. In this painful way he paſſed three years, when, upon being ſearched, a ſtone was felt in the bladder, and he ſubmitted to the operation. A ſtone of a deep mulberry colour, weighing an ounce and and a quarter, was extracted. The operation was fucceeded by violent inflammation, and he died on the third day.

He had expreffed a defire that his body might be opened after death, and it was accordingly examined the day following. The principal feat of the difeafe, as was expected, was found to be in the bladder, the whole of which was preternaturally thickened, and on its inner furface, about an inch from its neck, was obferved a confiderable fungus. The kidnies were flightly difeafed. The abdominal and thoracic vifcera afforded no remarkable appearance.

From what was obferved it feemed probable that at firft the ftone had adhered to the bladder, and occafioned little or no inconvenience till it was feparated by the violent exertion above fpoken of, and then gave rife to the formation of the fungus.

*Newman-ftreet,*

March 26, 1782.

